# Belowground productivity varies by assessment technique, vegetation type, and nutrient availability in tidal freshwater forested wetlands transitioning to marsh

**DOI:** 10.1371/journal.pone.0253554

**Published:** 2021-07-16

**Authors:** Andrew S. From, Ken W. Krauss, Gregory B. Noe, Nicole Cormier, Camille L. Stagg, Rebecca F. Moss, Julie L. Whitbeck

**Affiliations:** 1 U.S. Geological Survey, Wetland and Aquatic Research Center, Lafayette, Louisiana, United States of America; 2 U.S. Geological Survey, Florence Bascom Geoscience Center, Reston, Virginia, United States of America; 3 Department of Earth and Environmental Sciences, Macquarie University, Sydney, New South Wales, Australia; 4 National Park Service, Jean Lafitte National Historical Park and Preserve, New Orleans, Louisiana, United States of America; Shandong University, CHINA

## Abstract

Wetlands along upper estuaries are characterized by dynamic transitions between forested and herbaceous communities (marsh) as salinity, hydroperiod, and nutrients change. The importance of belowground net primary productivity (BNPP) associated with fine and coarse root growth also changes but remains the dominant component of overall productivity in these important blue carbon wetlands. Appropriate BNPP assessment techniques to use in various tidal wetlands are not well-defined, and could make a difference in BNPP estimation. We hypothesized that different BNPP techniques applied among tidal wetlands differ in estimation of BNPP and possibly also correlate differently with porewater nutrient concentrations. We compare 6-month and 12-month root ingrowth, serial soil coring techniques utilizing two different calculations, and a mass balance approach (TBCA, Total Belowground Carbon Allocation) among four tidal wetland types along each of two river systems transitioning from freshwater forest to marsh. Median values of BNPP were 266 to 2946 g/m^2^/year among all techniques used, with lower BNPP estimation from root ingrowth cores and TBCA (266–416 g/m^2^/year), and higher BNPP estimation from serial coring of standing crop root biomass (using Smalley and Max-Min calculation methods) (2336–2946 g/m^2^/year). Root turnover (or longevity) to a soil depth of 30 cm was 2.2/year (1.3 years), 2.7/year (1.1 years), 4.5/year (0.9 years), and 1.2/year (2.6 years), respectively, for Upper Forest, Middle Forest, Lower Forest, and Marsh. Marsh had greater root biomass and BNPP, with slower root turnover (greater root longevity) versus forested wetlands. Soil porewater concentrations of NH_3_ and reactive phosphorus stimulated BNPP in the marsh when assessed with short-deployment BNPP techniques, indicating that pulses of mineralized nutrients may stimulate BNPP to facilitate marsh replacement of forested wetlands. Overall, ingrowth techniques appeared to represent forested wetland BNPP adequately, while serial coring may be necessary to represent herbaceous plant BNPP from rhizomes as marshes replace forested wetlands.

## Introduction

We anticipate that carbon (C) sequestration will increase in tidal freshwater and low-salinity wetlands with greater organic matter production as atmospheric CO_2_ concentrations continue to climb [1]. Whether the organic matter is stored long-term is determined in part by coincident drivers of tidal wetland loss, including human-derived stressors and natural processes that dictate wetland transience [2, 3]. Storage of aboveground organic matter in tidal wetlands is generally regarded as being somewhat ephemeral; subjected to annual cycles of natural senescence in herbaceous wetlands or to decadal cycles of periodic disturbance in forested wetlands. In some cases, disturbance is infrequent enough in forested wetlands to assume longer-term accumulation of aboveground organic matter, and in those cases, biomass accumulation can be large [4]. Even so, the aboveground organic matter pool is subject to massive change in any given year [e.g., 5, 6].

As a consequence, organic matter accumulation below the soil surface in the form of live and dead root biomass, allochthonous and autochthonous organic and inorganic matter deposition, and algal/microbial growth provide the greatest potential for the long-term storage (> 100 years) of C-rich organic matter in tidal wetlands. In some cases, natural environmental change can even facilitate an increase in belowground organic matter accumulation. For example, Rogers et al. [7] reported greater soil organic matter accumulation in tidal wetlands of the Americas versus Australasia in response to higher rates of sea-level rise inundation over the Holocene, which stimulated root productivity and reduced decomposition versus similarly positioned tidal wetlands in Australasia. To avoid inundation beyond plant tolerance, tidal wetlands must increase soil surface elevation as sea levels rise [8–10]. Much of tidal wetland vertical soil expansion is associated with the accumulation of organic matter below the soil surface from root growth, or belowground net primary productivity (BNPP) [11].

Interest in BNPP studies in tidal wetlands have been inspired by the wide realization that tidal wetlands store large amounts of organic matter as roots in soil biomass [e.g., 12–14], which has prompted refinement of assessment techniques, further consideration of assumptions and variability in assessments from specific habitats, and extrapolations of soil biomass to the global scale [e.g., 15, 16]. BNPP technique assessment has received some attention in terrestrial ecosystems [17], but relatively little attention has been directed toward which technique may be best suited to assess BNPP in tidal wetlands.

Several past efforts have been made to understand environmental controls over BNPP in tidal wetlands, e.g. [18], including tidal freshwater forested wetlands (TFFW) of the Southeastern United States [19]. As TFFW transition to marsh through progressive salinization driven by human influence, sea-level rise, and climate change (e.g., drought) [20], there is an acute interest in determining how BNPP shifts with transformation to marsh [2, 14], and among low-salinity wetlands, the role of nutrient pulses in influencing BNPP. BNPP estimation and linkages to various drivers in low-salinity tidal wetlands may also be due to choice of estimation method that include different time frames or root types as plant growth form changes with transition from forest to marsh.

In this study, we first compared among BNPP estimation techniques and provide BNPP, root turnover, and root longevity estimates by habitat type for low-salinity tidal wetlands. Second, we explore how BNPP may be controlled by subtle changes in porewater nutrients as TFFW transition to marsh along salinity and hydroperiod gradients. We measured BNPP from upper estuarine locations along the Waccamaw and Sampit Rivers (South Carolina, USA) and Savannah River (Georgia, USA) using 6-month and 12-month root ingrowth assays [21], serial soil coring techniques utilizing two different BNPP calculation methods [22, 23], and a mass balance approach [24] deployed on the same sites and across different time scales.

## Materials and methods

### Ethics statement

The U.S. Fish and Wildlife Service granted permissions to conduct research on Savannah National Wildlife Refuge (Georgia, USA) and Waccamaw National Wildlife Refuge (South Carolina, USA).

### Study sites

**Fig 1:**
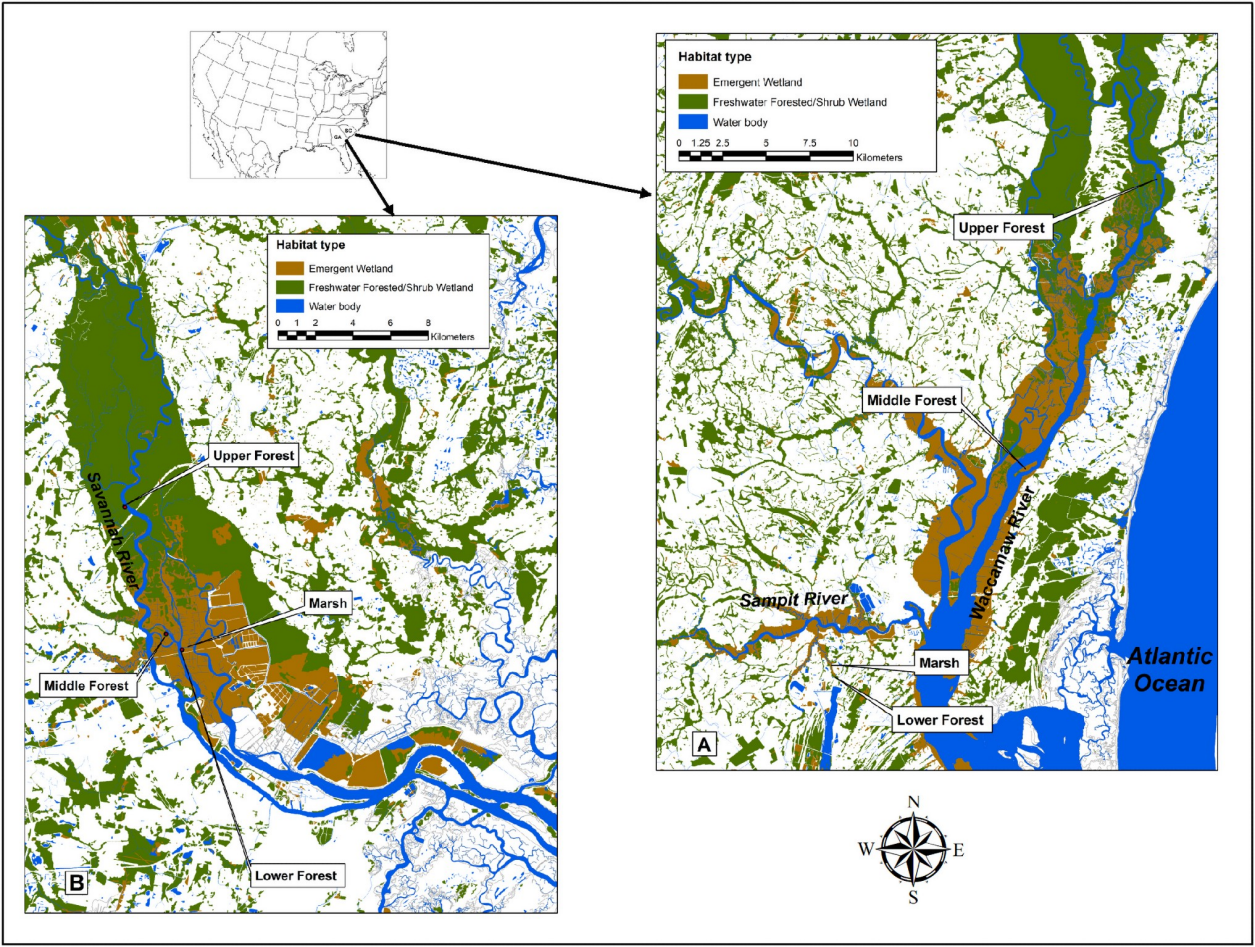
Location of study sites along the Waccamaw/Sampit Rivers, South Carolina, and Savannah River, Georgia. Sites represent tidal freshwater forested wetlands (TFFW, Upper Forest), TFFW experiencing low concentrations of salinity incursion (Middle Forest), former TFFW that is undergoing active transition to marsh from salinity intrusion (Lower Forest), and low-salinity herbaceous marsh. Wetland classifications were downloaded at, https://fwsprimary.wim.usgs.gov/wetlands/apps/wetlands-mapper/ (accessed 12 August 2020); however, the freshwater designation was removed from “emergent wetland” because current salinities are above 0.5 psu for much of this emergent wetland area. The image was created using ArcMAP (version 10.7) with the North America political boundaries from ScienceBase (Public Domain) at, https://www.sciencebase.gov/catalog/item/4fb555ebe4b04cb937751db9 (accessed 23 April 2021).

We established two transects along the lower coastal portions of the Waccamaw River and Sampit River in South Carolina and along the Savannah River in Georgia, both located along the Atlantic coast of the Southeastern United States (US) (Fig 1). These rivers all drain into estuarine embayments that eventually feed into the Atlantic Ocean. Each transect is composed of four sites: a TFFW (Upper Forest), a TFFW experiencing low concentrations of salinity incursion (Middle Forest), a TFFW that is undergoing active transition to marsh from salinity intrusion (Lower Forest), and a low-salinity herbaceous wetland (Marsh) [25].

All sites experience regular surface flooding from astronomical tides [20, 26]. Twelve-year monthly porewater salinity for Upper Forest sites averaged 0.1 psu, while salinity was 1.2–1.4 psu for Middle Forest sites, 2.4–4.3 psu for Lower Forest sites, and 3.1–4.9 psu for Marsh sites [14]. Periodic low-rainfall periods pulse salinity to these sites such that individual months can register considerable salinization [27]. Tidal range at the mouth of the Waccamaw/Sampit Rivers and Savannah Rivers is 1.1 m and 2.0 m, respectively, but channel morphology can amplify tidal range on specific sites. For example, the Marsh site along the Waccamaw/Sampit Rivers is flooded approximately 58 times/month by distinct tidal pulses, while the Upper Forest site is flooded approximately 38 times/month [28] (S1 and S2 Figs); not all tidal floods ebb with full de-watering of the depressional areas between trees (hollows). Upper Forests are inundated less with lower maximum flood depths than marshes, with Middle and Lower Forests having intermediate flood frequencies, durations, and depths [20]. All soils were classified as Typic Hydraquent, and were either of the levy series (Savannah Upper Forest; Waccamaw Upper and Middle Forests) or tidal marsh (fresh) series [29]. Soil bulk densities, phosphorus concentrations, and nitrogen concentrations were 0.19–0.30 g/cm^3^, 6.4–19.8 mmol P/cm^3^, and 0.09–0.13 mmol N/cm^3^, respectively, among the 8 study sites [30]. Soils were deep, averaging 11.4 m to benchmark rod refusal [31], and at a depth of 0.5 m, soil organic matter was 144 to >1000 years old across all sites [14], with most of the new root growth occurring at shallower soil depths (< 0.3 m) [32].

The dominant forest vegetation on Upper Forest sites (88–93% tree canopy cover) includes baldcypress (*Taxodium distichum*), water tupelo (*Nyssa aquatica*), and swamp tupelo (*Nyssa biflora*), as well as some individual red maple (*Acer rubrum*), ash (*Fraxinus* spp.), and oak (*Quercus* spp.). As salinity concentrations increase, Middle Forest sites (75% tree canopy cover) typically include only baldcypress with some swamp tupelo and tag alder (*Alnus serrulata*), and Lower Forest sites (8–14% tree canopy cover) have a mix of live and dead baldcypress along with encroaching marsh species representative of the adjacent marsh sites. Marsh sites (0% tree canopy cover) include *Spartina cynosuroides*, *Schoenoplectus robustus*, *Typha latifolia*, *Scirpus robustus*, and *Zizaniopsis miliacea*, and a suite of other marsh species of lesser occurrence. Importantly, the herbaceous understory cover increases as the forest canopy breaks up with transgression from freshwater Upper Forest to low-salinity Marsh such that herbaceous marsh species are also an important component of Lower Forest sites.

### Root biomass standing stock

Belowground biomass was collected from 20 soil cores harvested from each site at the beginning of the growing season in 2016 (160 cores) and from 10 cores collected from each site in 2017 (80 cores). On the forest sites, soil cores were extracted approximately 1 m from a co-dominant baldcypress tree for standardization. On the marsh sites, soil cores were extracted 5 m apart in a straight line approximately perpendicular to the river channel.

We used a 5-cm-diameter, thin-walled, stainless steel coring device with a beveled base for cutting. The device was hammered into the soil on all sites to approximately 30 cm with minimal compaction (<0.5 cm). When lower portions of the sample did not accompany the extracted core, we extracted them using a separate modified PVC pipe of identical dimensions but with a cap that facilitated suction extraction, similar to a piston corer. Cores were extracted, and then sectioned at soil depths of 0–10 cm, 10.1–20 cm, and 20.1–30 cm. Sections were placed in pre-labeled plastic bags, transported from the field on ice, and kept refrigerated at 4°C until processing.

Soil core sections (N = 720 total) were washed over a 5-mm sieve to remove loose soil material but retain organic matter. The remaining contents of each core were deposited individually into trays of fresh deionized water, and plant material was separated visually. Live roots were distinguishable from dead roots by being translucent and light in color (white to tan, as per [33]) and live roots were buoyant. In addition, dead roots appeared as described by McKee et al. [10] as displaying “…loss of structural integrity, colour [sic] and signs of decomposition”. Sieved material from each soil core section was further separated into categories of live fine roots (≤ 2 mm diameter), live coarse roots (>2 mm diameter, incl. rhizomes), and root necromass (dead roots, all size classes), as well as “other organic matter” (e.g., detrital bark, wood, plant structures, leaves). While our definition of fine root is ≤ 2 mm diameter, “fine” has been variously defined in the literature as ranging from < 1 mm to upwards of 5 mm diameter [34]. The 2-mm designation in dividing fine from coarse roots was a natural division since roots larger than this usually contain secondary xylem thickening and tend to be perennial [33, 35]. After separations were completed, all material was dried to a constant mass at 70 °C. Root biomass (kg m^-2^) was summed over the 30-cm depth by category (fine root, coarse root, necromass, other organic matter), and scaled to 1 m^2^ based on the surface area of the core.

### Root ingrowth assay

Root ingrowth techniques follow those introduced by Raich et al. [21] for terrestrial forests, and these techniques since have been applied widely, including to tidal wetlands [e.g., 10, 36, 37]. We assessed growing season root ingrowth over 12-month and 6-month periods, *via* experiments which we initiated at the start of the growing season in March 2016 and March 2017, respectively. A total of 10 ingrowth cores were deployed on each site in 2016 (total of 80 cores) and left in place for 12 months, and an additional 10 cores were deployed on each site in 2017 (total of 80 cores) and left in place for almost 7 months to complete the growing season on these sites, e.g., [26].

For each assessment period, we used the same holes (voids) that were created during soil extraction for root biomass standing stock described in the previous section. After biomass core extraction, we immediately replaced voids with ingrowth bags of the same dimensions as the voids (5-cm diameter x 30 cm). Ingrowth bags were made of synthetic plastic, flexible mesh with ~1 mm apertures that allow roots to grow into bags without resistance [10, 36]. Approximately 750 cm^3^ of dry, commercially available peat moss (Promix fine grain *Sphagnum*, Premier Tech Horticultural, Inc., Quakertown, PA, USA) was combined with 225 cm^3^ of sand, and wet with ~300 cm^3^ of water to fit in each 589 cm^3^ ingrowth core. The ratio of peat moss and sand was chosen to approximate the upper soil compositions of these sites based on past study [2, 20], to standardize the ingrowth material, and to ensure that soil density did not impede root penetration into the inert material. Root ingrowth bags were sealed with plastic cable ties once filled, and each was attached with a numbered aluminum tag for cross-referencing during extraction.

After 12 and 6 months of deployment, ingrowth cores were extracted individually using a serrated knife to carefully sever all root material from around the perimeter of each bag. Bags were gently lifted, cut into sections (0–10, 10.1–20, 20.1–30 cm), and immediately placed in individual plastic bags for transport from the field on ice until they were placed in a refrigerator. Ingrowth cores were processed identically to soil cores, except that once washed over a 5 mm sieve, organic material comprised only roots (live fine root biomass, live coarse root biomass, root necromass), which was distinguishable from the composite *Sphagnum* filler material we used. After separations were completed, all material was dried to a constant mass at 70 °C.

Two 6-month cores from the Savannah Upper Forest and four 6-month cores from the Savannah Middle Forest were damaged during processing and were omitted from the analysis. BNPP calculations using ingrowth of new roots over the respective deployment periods were determined by summing all live fine root, live coarse root, and root necromass, since necromass represents roots that grew into the cores but died and began to decompose over the period of deployment.

### Serial coring techniques

Serial cores were collected and handled using similar techniques described above for initial root biomass standing stock sampling. Serial cores were collected six times over 1.5 years (March 2016, June 2016, September 2016, December 2016, March 2017, October 2017), for five growth intervals. Ten, 5-cm-diameter cores were collected at each site during each sampling event, but unlike root biomass standing stock cores, we sectioned samples into two depth intervals (0–10 cm, 10.1–30 cm) instead of three. This was necessary because we could not prevent mixing of the 10.1–30 cm depths when sites were flooded deeply.

For each serial core, we separated live fine roots, live coarse roots, and root necromass, drying biomass to a constant mass at 70 °C. We used two calculation methods to determine BNPP. The first calculation method is most often referred to as the Smalley method [38] and is very similar in application to the “Decision Matrix” approach described for forest BNPP calculations using serial coring [23]. Stagg et al. [39] provided a meta-analysis of BNPP estimation from the Smalley method for coastal wetlands, and describe its formulation as follows:

$$dB_{\text{t}\left(\text{i}+1\right)-\text{ti}}=\left(B_{\text{live},\text{t}(\text{i}+1)}–B_{\text{live},\text{ti}}\right)+\left(B_{\text{dead},\text{t}(\text{i}+1)}–B_{\text{dead},\text{ti}}\right)$$ (1)

where *dB*_t(i+1)-ti_ is the live (*B*_live_) and dead biomass (*B*_dead_) between any two sampling events (t(i+1)-ti), which are summed through a series of decision steps outlined comprehensively in [23] to produce estimates of BNPP, as follows:

$$\text{BNPP}=\sum_{i=1}^{T-1}dB_{t\left(i+1\right)-\text{ti}}$$ (2)

where *T* is the total number of sampling events, in our case six. We had no missing data among these six serial coring dates. This technique sums all changes in live root biomass and dead root necromass.

The second calculation method, known as the Maximum-Minimum (Max-Min) method [34] does not account for root mortality among serial coring dates, and thus ignores necromass dynamics. This method subtracts the lowest live root biomass value (*B*_live, lowest_) of each individual core from the highest live root biomass value (*B*_live, highest_) of each individual core from individual replicate serial coring locations, without regard to when those values occurred from that location over the period of serial coring, as follows:

$$\text{BNPP}=B_{\text{live},\text{highest}}–B_{\text{live},\text{lowest}}$$ (3)

All serial core calculations of BNPP were adjusted to annual rates from cores harvested over 1.5 years.

### Mass balance approach

Total Belowground Carbon Allocation (TBCA) [24] was used to estimate BNPP through difference calculation based on the following estimates from our study sites [see 14]: soil surface CO_2_ flux (*F*_s_); C erosion, export, and CH_4_ efflux (*F*_E_); C inputs from aboveground leaf, fruit, and twig litter associated with above ground litterfall (*F*_A_); change in C content of root biomass (coarse + fine) (*d*C_R_); and change in C content of the litter layer (*dC*_L_), using the following formula [24],

$$\text{TBCA}=F_{\text{S}}+F_{\text{E}}–F_{\text{A}}+d\left[C_{\text{S}}+C_{\text{R}}+C_{\text{L}}\right]/dt$$ (4)

where the undefined component (*dC*_S_: change in C content of mineral soil) is assumed for TFFW and marsh habitat to be 0. Also, since this approach tracks C through the system, and not biomass, an appropriate conversion from mass C data to organic matter was made to result in BNPP, as follows,

$$\text{BNPP}=\text{TBCA}/\left(\%\text{C}/100\right)$$ (5)

Where %C is the site-specific C content for organic matter in roots, which ranges from 38.2 to 47.1% C in root organic matter among the eight Waccamaw/Sampit and Savannah River sites (Table 1). These conversions were determined as part of a root and litter decomposition study from these same sites [25]; native roots were excavated from each site, washed of soil, composited, dried, ground, and analyzed on a CN Elemental Analyzer (model Flash EA 1112, ThermoFinnigan, Wigan, UK).

**Table 1:** Site-specific carbon content (%) of roots.

River	Site	% C
Waccamaw/Sampit	Upper Forest	47.05
Waccamaw/Sampit	Middle Forest	45.71
Waccamaw/Sampit	Lower Forest	44.92
Waccamaw/Sampit	Marsh	43.45
Savannah	Upper Forest	38.21
Savannah	Middle Forest	42.28
Savannah	Lower Forest	40.67
Savannah	Marsh	43.02

Results of root carbon content for samples collected along the Waccamaw/Sampit Rivers (South Carolina, USA) and Savannah River (Georgia, USA).

### Root turnover and longevity

Turnover of all roots (fine and coarse) was estimated with the average standing stocks of live fine and live coarse roots (necromass was excluded) from root biomass standing stock cores taken in 2016 and 2017. Root turnover was calculated for 30-cm rooting depths as follows,

$$\text{Root}\;\text{turnover}=\text{BNPP}/B_{\text{ss}}$$ (6)

where BNPP (g/m^2^/year) is the rate of belowground net primary production, determined using each of the three estimation techniques or calculation methods that physically evaluated BNPP for up to a year or greater (12-month ingrowth, Smalley serial core estimation, Max-Min serial core estimation), and *B*_ss_ is mean live root standing stock biomass (g/m^2^), yielding units for root turnover of 1/year. This formula can be applied to fine roots only, as it is often done [40], or to roots of all size classes, as we do here. Root turnover describes how often roots are replaced on an annual basis; the lower the value, the slower the replacement of standing root volume by new roots. As such, root longevity is the inverse of root turnover, and is reported in years.

### Porewater nutrient sampling and analysis

We extracted soil porewater samples twice (September 2016; March 2017) from within 0.5 m of each ingrowth/serial core (N = 10 per site) using a porewater sipper tube [41]. A plastic tube was inserted to a soil depth of 15 cm (midpoint of 0–30 cm core), and porewater was extracted through Tygon tubing using a 60 mL syringe. Initial water drawn through the tubing was discarded and re-drawn until clarity improved. Once clear and void of large particles, water was collected and injected through a 0.2 μm syringe filter into a labelled 20 mL acid-washed plastic vial. Porewater samples were frozen, shipped, thawed and re-filtered (0.2 μm polyethersulfone), refrigerated, and analyzed within 48 hours. Samples were analyzed for dissolved reactive orthophosphate (DRP), nitrate (as NO_3_^-^ + NO_2_^-^, NO_x_), and ammonia (NH_3_) using a discrete analyzer (Seal AQ2, SEAL Analytical Inc., Mequon, Wisconsin, USA). Every discrete analyzer run included an external reference standard diluted to within the range of the porewater samples (QA/QC threshold of ± 10% of published value; ERA, Arvada, Colorado, USA).

### Statistical analyses

First, we applied ANOVA techniques to identify differences among rivers and sites in biomass of live fine roots, live coarse roots, necromass, and other organic matter from the standing stock root biomass cores extracted in 2016 and 2017. Second, we used correlation analysis (Pearson’s r) to explore relationships between each of the four BNPP techniques/calculations, which included (1) 6-month ingrowth, (2) 12-month ingrowth, (3) Smalley serial core calculations, and (4) Max-Min serial core calculations, and porewater nutrient concentrations (DRP, N0_x_, NH_3_). Since all of these data collections were co-located, up to 10 comparisons were possible for each site; 40 per river basin (N = 80). TBCA estimations of BNPP were not considered statistically because they were not replicated by site and replicated only once by river basin.

Third, we tested our overall hypothesis that BNPP estimates differed among technique. Exploratory correlation analysis suggested that techniques were not correlated across all sites (p<0.05). However, when explored across all sites, data from each technique were non-normal and could not be fully transformed (i.e., data remained heteroscedastic). In order to test this hypothesis properly across all sites, we first used non-parametric statistics, Proc NPAR1WAY (Wilcoxon signed-rank test), to determine if differences existed among the techniques used across all sites. We then used ANOVA techniques on the best-transformed data to evaluate interactions (technique|river|site).

Fourth, we used mixed-model ANOVA to determine if BNPP estimation varied by river and site, and when significant (α = 0.05), we applied a Tukey’s test with Bonferroni adjustment against mean values to determine how differences distributed among technique by individual site. Normalization and homogeneity of variance were attainable through linear rank transformations within a BNPP technique. While not part of study results per se, we applied a multiple regression approach to help frame our discussion of relationships between herbaceous aboveground and total aboveground productivity from past study and the different empirical estimates of BNPP.

We used an alpha of 0.05 for all hypothesis testing associated with correlation analyses, non-parametric analysis, ANOVAs, and regression. When data were transformed, a natural log transformation (Ln + 1) was most successful. Means ± 1 standard error (SE) are reported throughout. All analyses were conducted using SAS 9.4 Software (SAS Institute, Inc., Cary, NC, USA).

## Results

### Distribution of root biomass among sites (standing stocks)

Live fine root biomass to a soil depth of 30 cm was 0.17–2.62 kg m^-2^ and live coarse root biomass was 0.24–1.58 kg m^-2^ among the eight sites across two years (Table 2). The ranking of biomass differences among sites along a river, from Upper Forest to Marsh, did not change significantly between the two rivers for 2016, as indicated by non-significant river × site interactions for both live fine and coarse root biomass, but did for 2017. Salinization mattered, such that transition to full marsh facilitated a 2.8–6.1 fold increase in live fine root biomass and a 1.6–6.6 fold increase in live coarse root biomass relative to freshwater forests along each river, as plant communities changed from woody to herbaceous. Live roots represented 73% of the biomass of total dead roots on Upper and Middle Forest sites, 112% on Lower Forest sites as marsh plants encroached (especially on the Savannah Lower Forest), and 460% on marsh sites, suggesting a more prominent role for live root versus dead root biomass in marshes. However, this average percentage in marshes was biased by an approximate 1000% live-to-dead root ratio for Waccamaw Marsh in 2017. Otherwise, the average live-to-dead root ratio was 279% live for Marsh sites. Other organic matter averaged 0.51–8.76 kg m^-2^ among all sites (Table 2), and tended to be lower on marsh sites having limited sources of woody debris.

**Table 2:** Characteristics of the root zone to a depth of 30-cm in 2016 and 2017.

	Waccamaw River				Savannah River				N_cores_	*F-*ratio_Site_	*P*_*Site*_	*P*_River x SIte_
**2016**	**Upper Forest**	**Middle Forest**	**Lower Forest**	**Marsh**	**Upper Forest**	**Middle Forest**	**Lower Forest**	**Marsh**				
Fine root (≤ 2 mm)	0.29 ± 0.04	0.49 ± 0.05	0.27 ± 0.04	1.64 ± 0.20	0.49 ± 0.17	0.26 ± 0.03	0.35 ± 0.08	1.36 ± 0.20	20	26.97	<0.0001	ns
Coarse root (> 2 mm)	0.24 ± 0.04	0.41 ± 0.06	0.44 ± 0.18	1.58 ± 0.33	0.82 ± 0.32	0.32 ± 0.08	0.61 ± 0.16	1.30 ± 0.25	20	8.92	<0.0001	ns
Dead roots (necromass)	1.20 ± 0.21	2.15 ± 0.27	2.05 ± 0.36	1.10 ± 0.22	0.79 ± 0.12	1.37 ± 0.16	0.67 ± 0.15	1.00 ± 0.23	20	108.6	<0.0001	<0.0001
Other organic matter	5.57 ± 0.51	8.76 ± 0.50	6.28 ± 0.62	2.03 ± 0.19	3.31 ± 0.34	8.34 ± 0.84	2.12 ± 0.26	1.03 ± 0.12	20	5.13	0.0029	0.0297
**2017**	**Upper Forest**	**Middle Forest**	**Lower Forest**	**Marsh**	**Upper Forest**	**Middle Forest**	**Lower Forest**	**Marsh**				
Fine root (≤ 2 mm)	0.46 ± 0.07	0.41 ± 0.07	0.17 ± 0.03	2.62 ± 0.17	0.27 ± 0.04	0.50 ± 0.04	0.55 ± 0.05	1.64 ± 0.43	10	36.29	<0.0001	<0.0001
Coarse root (> 2 mm)	0.29 ± 0.06	0.54 ± 0.15	0.26 ± 0.11	1.54 ± 0.26	0.40 ± 0.09	0.68 ± 0.24	0.99 ± 0.22	1.39 ± 0.20	10	12.76	<0.0001	0.0088
Dead roots (necromass)	1.30 ± 0.18	1.42 ± 0.26	1.52 ± 0.35	0.40 ± 0.07	0.82 ± 0.13	1.36 ± 0.32	0.64 ± 0.13	1.09 ± 0.34	10	77.92	<0.0001	<0.0001
Other organic matter	6.23 ± 0.77	7.39 ± 0.75	7.70 ± 0.66	0.51 ± 0.09	3.87 ± 0.42	7.49 ± 0.77	2.81 ± 0.46	1.56 ± 0.32	10	4.25	0.008	0.0033
Fine root depth ratio*	0.335	0.474	0.556	0.291	0.398	0.502	0.336	0.203	30	--	--	--
Coarse root depth ratio**	0.413	0.485	0.333	0.286	0.267	0.337	0.164	0.405	30	--	--	--

Samples were collected from Upper Forest, Middle Forest, Lower Forest, and Marsh sites along the Waccamaw/Sampit Rivers (South Carolina, USA) and Savannah River (Georgia, USA). N represents the number of cores used to estimate root zone characteristics on each site. Root biomass standing stocks are reported as mean values in kg m-2 (± 1 standard error).

* = Fine root biomass at a depth of 0–10 cm versus all live fine roots from 0–30 cm as an average of both years

** = Coarse root biomass at a depth of 0–10 cm versus all live coarse roots from 0–30 cm as an average of both years

Among all live fine roots, 20–56% of biomass within the top 30 cm of soil occurred in the top 10 cm (Table 2). The range was 16–48% for live coarse roots for the same comparison. Live fine roots tended to be more equally distributed from the soil surface to a depth of 30 cm on the two pure marsh sites than on the six sites with trees.

### Differences among BNPP technique and calculation methods

**Fig 2:**
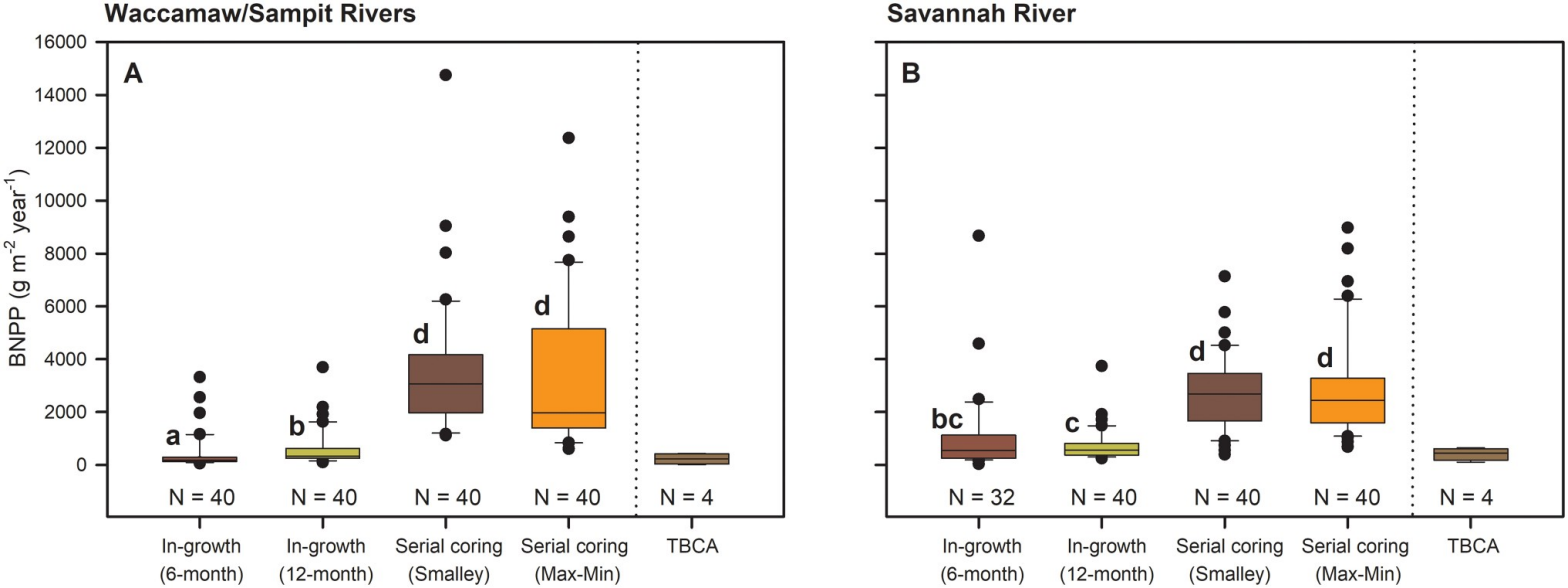
Belowground net primary productivity (BNPP, median) by river. (A) Waccamaw/Sampit Rivers, South Carolina; and (B) Savannah River, Georgia, as estimated using root ingrowth techniques over 6 months, root ingrowth techniques over 12 months, Smalley estimation of serial coring data, Max-Min estimation of serial coring data, and total belowground carbon allocation (TBCA) for data collected from TFFW transitioning to marsh. Mean values by technique and river followed by the same letter do not differ significantly (Technique × River × Site, n.s.; Technique × River, *F*_3,280_ = 9.77, *P* < 0.001).

**Fig 3:**
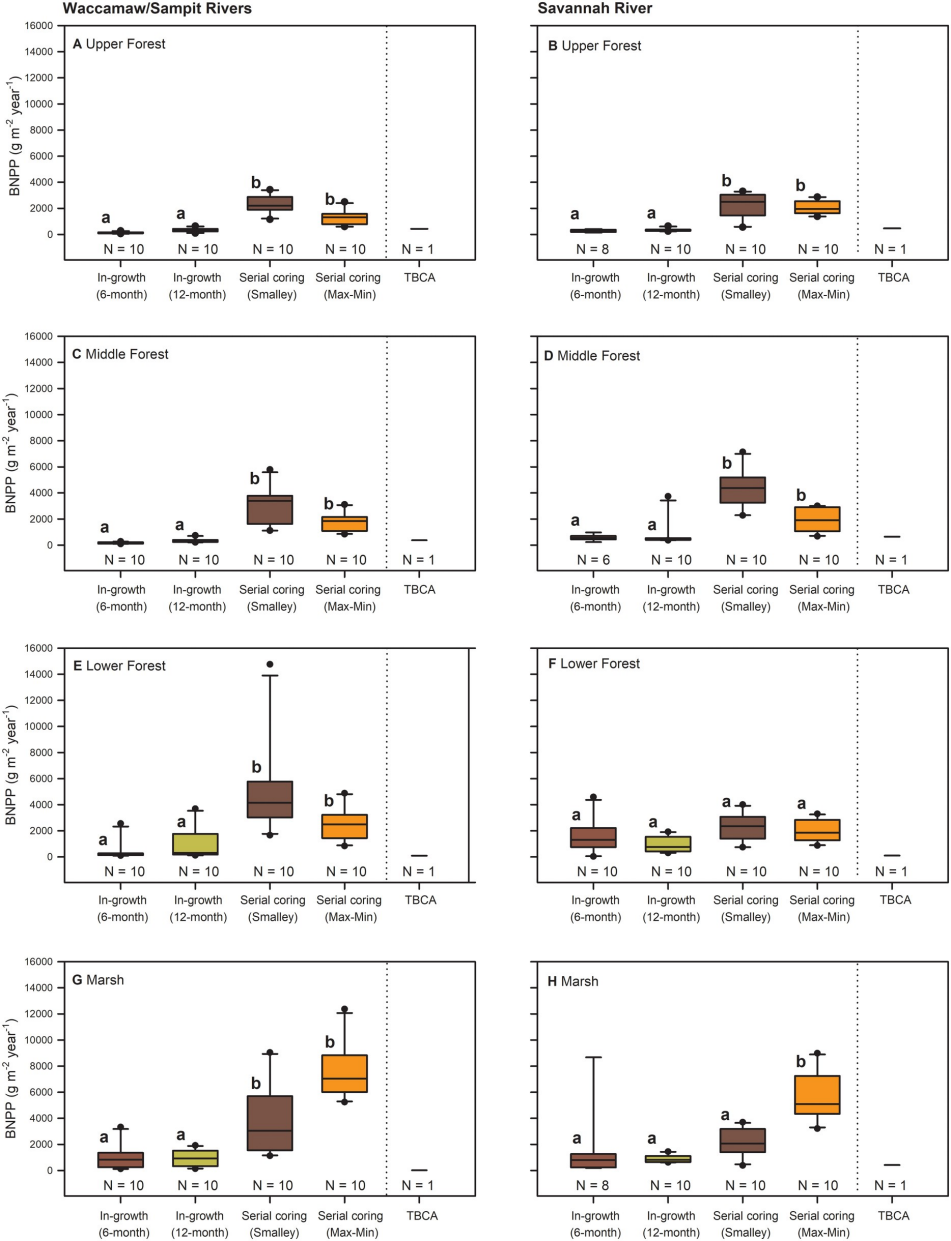
Belowground net primary productivity (BNPP, median) by technique and site for TFFW and marshes. BNPP was estimated using root ingrowth techniques over 6 months, root ingrowth techniques over 12 months, Smalley estimation of serial coring data, Maximum-Minimum (Max-Min) estimation of serial coring data, and total belowground carbon allocation (TBCA) for data collected from TFFW transitioning to marsh along the Waccamaw/Sampit Rivers, South Carolina, (A), Upper Forest, (C) Middle Forest, (E) Lower Forest, and (G) Marsh; and along the Savannah River, Georgia, (B), Upper Forest, (D) Middle Forest, (F) Lower Forest, and (H) Marsh. Mean values by technique and site followed by the same letter within a river do not differ significantly (Technique × River × Site, n.s.; Technique × Site, *F*_9,280_ = 4.91, *P* < 0.001).

Significant differences existed among BNPP technique or calculation method chosen (χ^2^ = 174.6; *P* < 0.001; df = 3), and these differences were affected by several high estimates of BNPP (> 8000 g/m^2^/year) from multiple individual cores (see S3 Fig to view across rivers). After data were ranked transformed, the ordering of these differences changed by river (technique × river; *F*_1,3_ = 5.39; *P* = 0.0013) (Fig 2) and by site (technique × site; *F*_7,21_ = 4.37; *P* < 0.0001) (Fig 3). Un-transformed data presented in box plots show how the different techniques estimate BNPP across all sites as well as the propensity for extreme values from individual cores. Serial coring, whether Smalley or Max-Min calculation methods are applied, provided the highest estimation of BNPP from our tidal wetlands, with median values among sites and cores of 2946 g/m^2^/year (range, 384 to 14749) and 2336 g/m^2^/year (range, 601 to 12366), respectively. Median estimates from 12-month ingrowth cores were 56% higher (at 416 g/m^2^/year; range, 96 to 3735) than median estimates from 6-month ingrowth cores (at 266 g/m^2^/year; range, 28 to 8671). For comparison, the median estimate for TBCA was 396 g/m^2^/year (range, 17 to 650). Technique × river and technique × site means are provided in Table 3.

**Table 3:** Belowground net primary productivity (BNPP) to a depth of 30-cm for TFFW and marshes by technique.

	Waccamaw River				Savannah River				N_num_;N_den_	*F-*ratio_Site_	*P*_*Site*_	*P*_River x Site_
Technique	Upper Forest	Middle Forest	Lower Forest	Marsh	Upper Forest	Middle Forest	Lower Forest	Marsh				
*In-growth (6-month), g m^-2^ year^-1^	140.8 ± 19.6	175.4 ± 20.4	418.5 ± 238.0	1016.4 ± 310.0	271.7 ± 30.0	571.5 ± 75.4	1584.5 ± 408.8	1649.5 ± 906.4	3; 64	7.12	0.0003	ns
In-growth (12 month), g m^-2^ year^-1^	333.7 ± 48.2	344.5 ± 49.5	922.8 ± 380.3	931.1 ± 206.9	358.5 ± 34.2	783.1 ± 328.7	920.3 ± 183.4	894.8 ± 91.7	3; 72	6.13	0.0009	ns
Serial coring (Smalley method), g m^-2^ year^-1^	2316.9 ± 664.1	3081.8 ± 1424.0	5122.7 ± 3649.2	3962.7 ± 2720.5	2252.1 ± 925.4	4322.5 ± 1461.5	2308.8 ± 1043.4	2185.7 ± 1046.9	3; 72	2.77	0.0478	0.0080
Serial coring (Max-Min method), g m^-2^ year^-1^	1310.8 ± 562.4	1796.0 ± 729.9	2511.1 ± 1279.7	7574.1 ± 2112.8	2051.3 ± 533.1	1942.4 ± 881.9	2062.6 ± 830.9	5686.1 ± 1874.5	3; 72	48.92	< 0.0001	0.0182
TBCA**, g m^-2^ year^-1^	431.5	374.1	97.1	17.3	470.3	650.0	107.7	416.6	--	--	--	--
AVERAGE, g m^-2^ year^-1^	906.7	1154.4	1814.4	2700.3	1080.8	1653.9	1396.8	2166.5	--	--	--	--

Samples were collected from Upper Forest, Middle Forest, Lower Forest, and Marsh sites along the Waccamaw/Sampit Rivers (South Carolina, USA) and Savannah River (Georgia, USA).

* Estimated over the approx. 6-month growing season.

** TBCA = Total Belowground Carbon Allocation.

For serial coring, two different patterns emerged. First, river x site interactions are significant (*P* ≤ 0.018), meaning that differences in BNPP among similarly positioned sites differ for serial coring estimation depending on the river (Fig 3). Second, estimation of BNPP using serial coring and two different calculation methods are ~7 times higher than for 6-month ingrowth and 5 times higher than 12-month ingrowth BNPP estimates, with discrepancies varying by site (Table 3). The Smalley method trended toward higher estimates of BNPP for forested sites versus all methods, while the Smalley method trended toward lower estimates of BNPP for the marsh sites versus the Max-Min method (Table 3, Fig 3). TBCA estimation of BNPP was most inconsistent with the others on sites with larger proportional amounts of herbaceous marsh plants along both rivers (Lower Forest and Marsh). However, as an average of all techniques, BNPP variation was more limited among sites: 907–1081 g/m^2^/year for freshwater Upper Forest sites, 1154–1654 g/m^2^/year for Middle Forest sites, 1397–1814 g/m^2^/year for Lower Forest sites, and 2167–2700 g/m^2^/year for Marsh sites.

### Porewater nutrients and BNPP relationships

**Fig 4:**
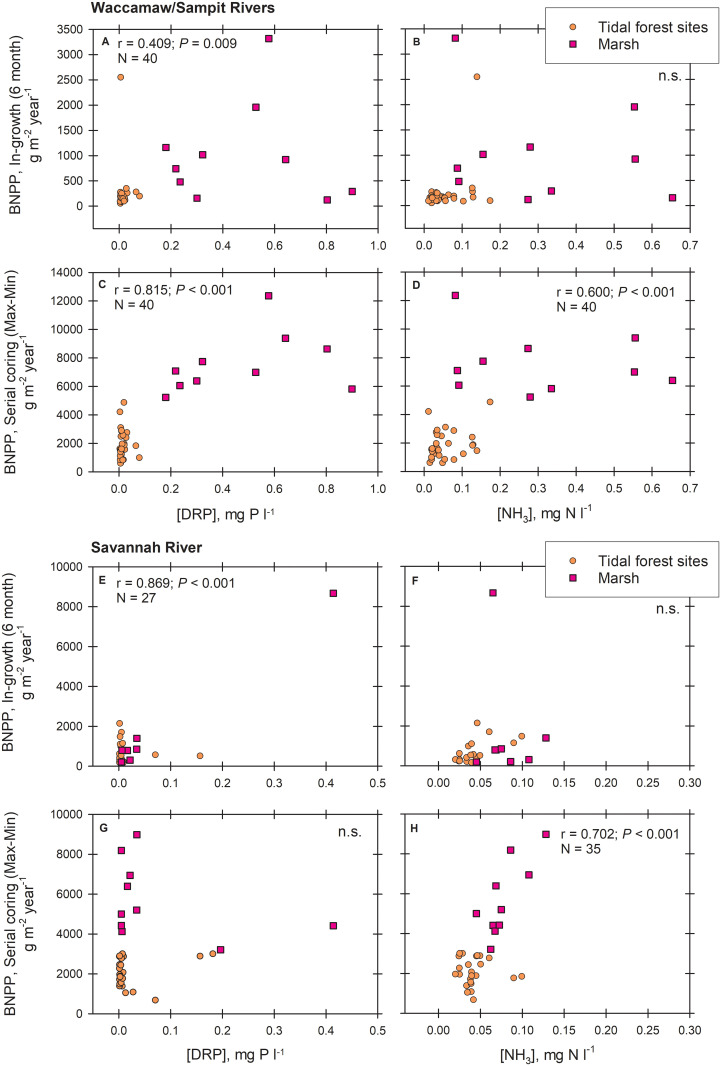
Correlation analyses of belowground net primary productivity (BNPP) versus porewater nutrient concentrations. Data are from the Waccamaw/Sampit Rivers, South Carolina, relating (A) 6-month ingrowth BNPP to Dissolved Reactive Phosphorus (DRP), (B) 6-month ingrowth BNPP to ammonia (NH_3_), (C) Max-Min serial coring BNPP to DRP, and (D) Max-Min serial coring BNPP to ammonia (NH_3_); and from the Savannah River, Georgia, relating (E) 6-month ingrowth BNPP to DRP, (F) 6-month ingrowth BNPP to ammonia (NH_3_), (G) Max-Min serial coring BNPP to DRP, and (H) Max-Min serial coring BNPP to ammonia (NH_3_) from TFFW transitioning to marsh. No significant relationships were found among porewater nutrients and other BNPP techniques used by site.

The most consistent relationships between BNPP estimation and porewater nutrients as an average of both sampling times happened when BNPP estimation occurred mathematically over short-term growth increments; 6-month ingrowth and Max-Min serial coring calculations (which sometimes use growth increments separated by only a couple of months). Specifically, 6-month ingrowth (Fig 4A) and Max-Min serial core estimation of BNPP (Fig 4C) related to DRP concentrations in the porewater across all Waccamaw/Sampit River sites (Pearson’s r = 0.41–0.82; *P* ≤ 0.009), but were driven strongly by high DRP concentrations in the marsh. BNPP estimates using 6-month ingrowth cores (Fig 4E) also related to DRP concentrations across all Savannah River sites (Pearson’s r = 0.87; *P* < 0.001), but with less prominence versus DRP concentrations in marsh sites. Furthermore, Max-Min serial core BNPP estimation related to porewater NH_3_ concentrations along both the Waccamaw/Sampit Rivers (Fig 4D; Pearson’s r = 0.60; *P* < 0.001) and Savannah River (Fig 4H; Pearson’s r = 0.70; *P* < 0.001), but 6-month ingrowth BNPP estimation did not (Fig 4B and 4F). Higher concentrations of NH_3_ occurred consistently in the marsh along the Waccamaw/Sampit Rivers, influencing those correlations, but concentrations of NH_3_ were not consistently higher in Savannah’s marsh.

We found no relationships among porewater nutrients sampled and BNPP estimations for any of the forested sites when analyzed singularly. However, for the two marsh sites on each river, BNPP did relate to porewater nutrients. For the marsh sites along the Waccamaw/Sampit Rivers, both 6-month ingrowth and Max-Min BNPP related to NO_x_ (Pearson’s r = 0.80–0.82; ≤ 0.006), and along the Savannah River, 6-month ingrowth related to DRP (Pearson’s r = 0.99; *P* < 0.001) and Max-Min estimation related to NH_3_ (Pearson’s r = 0.79; *P* = 0.007). Thus, BNPP for marsh sites along both rivers was responsive to concentrations of some nutrients in soil porewaters.

### Distribution of root growth by depth into the soil

**Fig 5:**
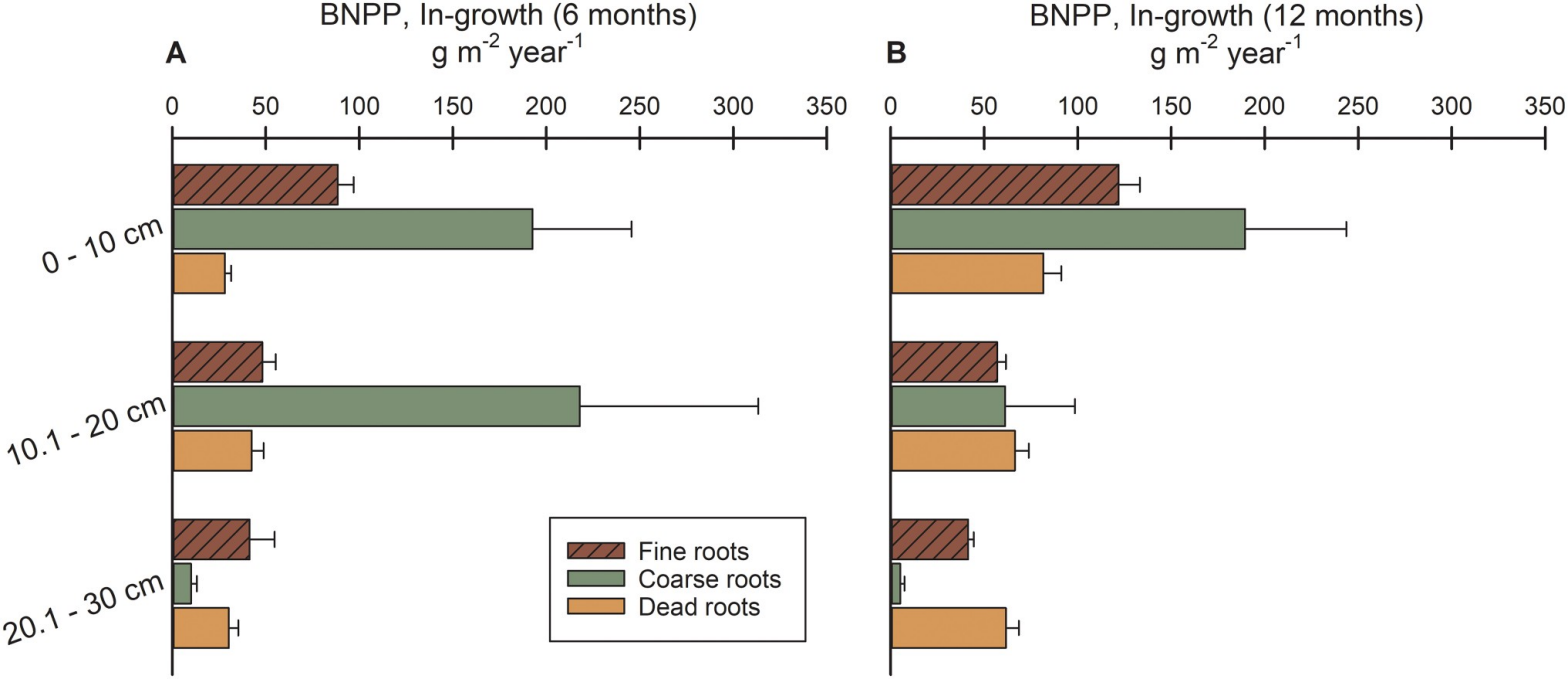
Belowground net primary productivity (BNPP) by sampling depth for TFFW and marsh using ingrowth. (A) 6-month ingrowth BNPP estimation by depth into the soil (0–10, 10.1–20, 20.1–30 cm) for fine roots (≤ 2 mm), coarse roots (> 2 mm), and dead roots (all diameters), and (B) 12-month ingrowth BNPP estimation by depth into the soil (0–10, 10.1–20, 20.1–30 cm) for fine roots (≤ 2 mm), coarse roots (> 2 mm), and dead roots (all diameters) for data collected from TFFW transitioning to marsh along the Waccamaw/Sampit Rivers, South Carolina, and the Savannah River, Georgia.

**Fig 6:**
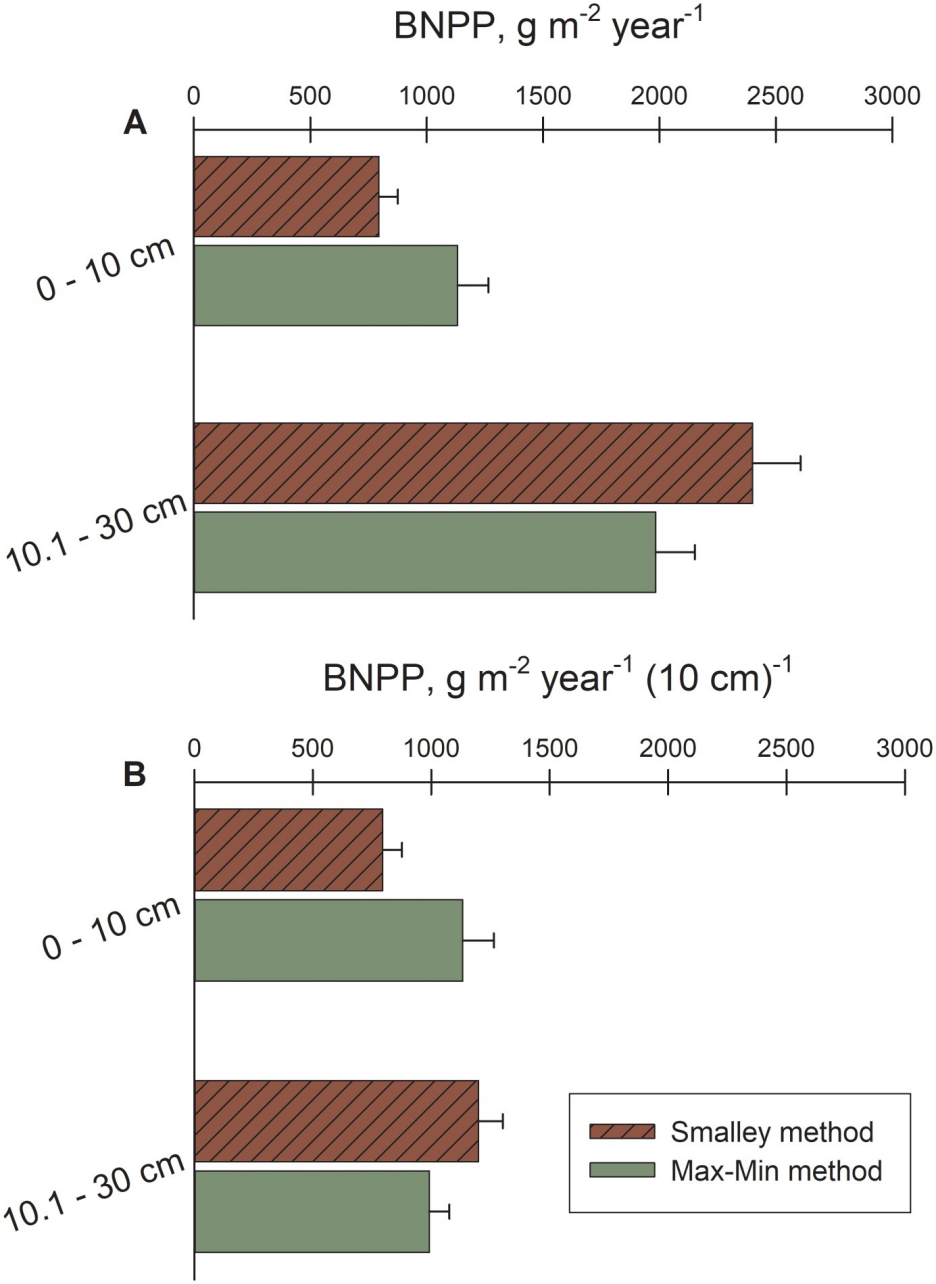
Belowground net primary productivity (BNPP) by sampling depth for TFFW and marsh using serial coring. (A) Smalley estimation and Max-Min estimation of belowground net primary productivity (BNPP) by depth into the soil (0–10, 10.1–30 cm) from serial cores, and (B) depth-adjusted Smalley estimation and Max-Min estimation of BNPP by depth into the soil (0–10, 10.1–30 cm) from serial cores from TFFW transitioning to marsh along the Waccamaw/Sampit Rivers, South Carolina, and the Savannah River, Georgia.

For both 6-month and 12-month ingrowth, BNPP at 0–10 cm depths was approximately 80% (Fig 5A) and 134% (Fig 5B) of BNPP at 10.1–30 cm depths, respectively, with proportionately smaller amounts of BNPP directed to 20.1–30 cm depths (3.8–5.3% of total). This is skewed slightly for 6-month ingrowth because single cores revealed a large growth increment for coarse roots (likely rhizomes) at a depth of 10.1–20 cm (Fig 5A). In contrast, for serial coring using both Smalley and Max-Min calculation methods, BNPP was greater at 10.1–30 cm depths versus 0–10 cm depths (Fig 6A). However, we had to pool these estimates at 10.1–30 cm depths because of our inability to clearly demarcate the separation between the two lower depths when pulling cores when sites were flooded. Thus, to account for the greater volumes represented in the first comparison (Fig 6A), we depth-standardized BNPP into 10-cm increments (Fig 6B). BNPP from serial coring indicated a more uniform distribution of root growth between the soil surface and 30 cm depths, although true sampling at 20.1–30 cm depths was not attained.

### Root turnover and longevity

As an average of all empirical techniques that physically assessed BNPP and included decomposition for up to one full year (i.e., excluding 6-month ingrowth and TBCA), live root turnover for Upper Forest sites averaged 2.2/year, meaning that the biomass of root size classes we quantified were replaced on this site 2.2 times in one year. Middle Forest sites averaged 2.7/year, Lower Forest sites averaged 4.5/year, and Marsh sites averaged 1.2/year (Table 4). Fine and coarse root longevity averaged 1.2–1.4 years for Upper Forest sites, 0.9–1.4 years for Middle Forest sites, 0.7–1.1 years for Lower Forest sites, and 2.0–3.2 years for marsh sites.

**Table 4:** Average root turnover (1/year) and longevity (years) to a depth of 30-cm for TFFW and marshes by technique.

	Waccamaw River				Savannah River			
**Turnover (1/year)**	**Upper Forest**	**Middle Forest**	**Lower Forest**	**Marsh**	**Upper Forest**	**Middle Forest**	**Lower Forest**	**Marsh**
In-growth (12 month)	0.604 ± 0.116	0.452 ± 0.104	3.225 ± 2.271	0.253 ± 0.056	0.522 ± 0.118	1.038 ± 0.462	0.773 ± 0.163	0.342 ± 0.047
Serial coring (Smalley method)	3.849 ± 0.411	4.331 ± 1.310	13.604 ± 4.077	1.105 ± 0.274	3.299 ± 0.706	5.643 ± 0.860	2.041 ± 0.410	0.921 ± 0.227
Serial coring (Max-Min method)	2.148 ± 0.297	2.340 ± 0.609	5.384 ± 0.981	2.166 ± 0.292	2.871 ± 0.596	2.494 ± 0.466	1.788 ± 0.290	2.203 ± 0.331
Average	2.200	2.374	7.404	1.175	2.231	3.058	1.534	1.155
**Longevity (years)**	**Upper Forest**	**Middle Forest**	**Lower Forest**	**Marsh**	**Upper Forest**	**Middle Forest**	**Lower Forest**	**Marsh**
In-growth (12 month)	2.76 ± 0.87	3.20 ± 0.53	1.74 ± 0.39	7.84 ± 2.48	2.95 ± 0.57	1.81 ± 0.36	1.81 ± 0.34	3.44 ± 0.45
Serial coring (Smalley method)	0.30 ± 0.04	0.44 ± 0.13	0.17 ± 0.06	1.37 ± 0.25	0.69 ± 0.28	0.22 ± 0.04	0.66 ± 0.11	2.09 ± 0.72
Serial coring (Max-Min method)	0.57 ± 0.09	0.60 ± 0.09	0.26 ± 0.04	0.52 ± 0.05	0.48 ± 0.07	0.54 ± 0.10	0.68 ± 0.09	0.55 ± 0.08
Average	1.21	1.41	0.72	3.24	1.37	0.86	1.05	2.03

Samples were collected from Upper Forest, Middle Forest, Lower Forest, and Marsh sites along the Waccamaw/Sampit Rivers (South Carolina, USA) and Savannah River (Georgia, USA).*

* Note that "In-growth (6 month)" was not included because a full year was not physically assessed, and TBCA (Total Belowground Carbon Allocation) was not included because of the lack of on-site replication for these calculations.

## Discussion

### Differences among BNPP techniques and calculation methods

Past studies outlined important disparities among techniques and calculation methods chosen in the assessment of BNPP for terrestrial forests [e.g., 23, 42]. Root ingrowth techniques were even cautioned for use as a sole, reliable BNPP technique until relationships with serial coring estimation were properly developed [42]. Thus, prior to initiating our study of BNPP in TFFW and marsh, we suspected that BNPP estimations might differ among the techniques and calculation methods that we chose because of disparities in inter-site plant community structure, morphology of vegetation, and potential artifacts imposed by specific BNPP procedures. We discovered that BNPP across all sites ranged from median values of 266 to 2946 g/m^2^/year, with serial core estimation relatively high (2336–2946 g/m^2^/year), and ingrowth core (266–416 g/m^2^/year) and TBCA estimation (396 g/m^2^/year) relatively lower.

To expand on potential technique differences, a modeling approach was used to sort among BNPP techniques for temperate forests [43]. Both the “decision matrix” (i.e., Smalley calculation method in our study) and Max-Min calculation methods were modelled, and compared with a compartment flow, or budget approach, similar to what was applied as a standard for comparison by Nadelhoffer and Raich [34]. While not a perfect match, the compartment flow model chosen by Publicover and Vogt [43] also uses similar assumptions as our TBCA approach; including annualized root biomass loss in estimation of production [24]. The compartment flow approach was considered superior to serial core estimates using either calculation (decision matrix, Max-Min) because compartment flow was less sensitive to sampling interval and seasonally high or low biomass values that may be missed during serial core extraction [43]. From this perspective, estimates by root ingrowth appear stronger than serial coring of low-salinity tidal wetlands since they are in closer agreement with TBCA. However, minirhizotron techniques–which we did not evaluate in our study–were considered superior to serial coring, ingrowth, and TBCA approaches in long-leaf pine (*Pinus palustris*) and wiregrass (*Aristida beyrichiana*) [44]. BNPP estimation from minirhizotrons did correlate positively with TBCA and negatively to serial coring and root ingrowth [44]; therefore, it may not be prudent to dismiss TBCA (and its agreement with ingrowth in our tidal wetlands) as a reliable BNPP estimation technique for some wetland habitats. Further exploration of TBCA, including a better understanding of intra-site variation in tidal wetlands, is warranted.

In our study, serial coring estimated higher BNPP than root ingrowth regardless of the calculation method chosen (Smalley or Max-Min). Serial coring, root ingrowth, and TBCA techniques all included decomposition in some way by separating dead roots, allowing for *in-situ* decomposition before harvesting, or through compartment flow approaches. It is indeed tempting to suggest that root ingrowth and TBCA techniques are best because they come closer in estimation across most of our tidal wetlands. However, large disparities between root ingrowth and TBCA occurred on individual sites to warrant some caution (Fig 3), although high variability certainly reflects the true nature of how ecosystem-level root growth occurs spatially. TBCA integrates spatially more effectively than the other BNPP approaches, in that plots used for assessment of some components of TBCA spanned a continuous area of 0.1 ha [14]. Furthermore, root ingrowth techniques estimated BNPP more closely to TBCA for the fresher upper and middle forest sites with greater microtopography (e.g., hummocks and hollows) than for more saline lower forest and marsh sites where more herbaceous and rhizomatous vegetation was present (Table 3, Fig 3). Serial coring techniques overestimate BNPP relative to TBCA by a considerable amount with consistency across all sites, but especially in marsh environments possibly because of the periodic inclusion of rhizomes.

In determining an appropriate BNPP technique for transitional wetland habitats, bias associated with the form and function of root growth characteristics among comparative sites can certainly occur [45]. Serial coring techniques likely provide reasonable estimation of BNPP if the intent is also to include rhizomes because rhizome growth is not affected by sampling techniques as it is for ingrowth. High estimates of BNPP using this approach in some marshes are likely real differences related to rhizomes. For example, BNPP from a range of freshwater-to-brackish marshes across the United States, Italy, and Canada–as determined using serial cores (Smalley calculations)–was as high as 7300 g/m^2^/year for a brackish *Spartina patens* marsh in Louisiana, USA [39]. Our estimates of BNPP using Smalley calculation methods were 3074 g/m^2^/year for marshes, but averaged 6630 g/m^2^/year for marshes using Max-Min calculation methods (Table 3), where rhizome activity could be influencing estimates because of high maximum standing root biomass at specific times. In contrast, cutting through large roots and small rhizomes, and inserting root-free material during ingrowth bag installation likely affect the larger versus finer roots of ingrowth cores. Fine roots were quicker to recover during forest re-growth after damage from tree cutting than were coarse roots in subtropical humid forests of India [46], and thus coarse root recovery may lag and be less represented by ingrowth BNPP. Yet, since only approximately 50% of root biomass standing stocks were in the coarse fraction across all of our sites (Table 2), this root size explanation may not explain all differences between ingrowth and serial coring.

Finally, when root growth with depth is compared using ingrowth (6 month and 12 month), at least half of fine root BNPP occurred in the upper 10 cm of all sites (Fig 5A and 5B). Greater than 90% of all roots were within the upper 30 cm of the soil profile among a variety of natural ecosystems surveyed globally [47], and 63% and 83% of roots were in the upper 30 cm for temperate deciduous forests and temperate grasslands, respectively [45]. Vertical distributions of BNPP were not as clear for serial coring techniques, possibly explained by cores being fully occupied by existing root biomass between each sampling period versus roots being allowed to grow unimpeded into root-free soil volumes. Root-free versus root-occupied growth media may also affect turnover (Table 4), with slower turnover and greater root longevity among roots occupying new volumes (ingrowth) versus under competition (serial coring). However, approximately 101 g/m^2^ and 210 g/m^2^ of roots died over 6 and 12 months of ingrowth assays, respectively, suggesting that root mortality may still be fairly high by ingrowth assay but also realistic of what would happen naturally given the root turnover rates that we document.

### Root turnover, longevity and nutrient acquisition

Roots turned over between 1.2 to 7.4 times per year among our sites (Table 4), and the turnover rates were consistently less frequent when estimated from ingrowth (maximum value of 3.2/year) versus serial coring (maximum value of 13.6/year). Brunner et al. [23] estimated lower root turnover from European forests as 0.7 to 2.6/year using root ingrowth and 0.2 to 3.1/year using serial coring techniques. We also did not limit our calculations to just those roots ≤ 2 mm diameter, and included roots or small rhizomes as large as 5 mm in diameter, which have critical function in tidal wetlands in affecting soil surface elevation change in response to rising seas [7, 10]. A previous study on the same Waccamaw/Sampit River sites found root turnover to be 2.1 for Upper Forest, 1.8 for Middle Forest, 1.5 for Lower Forest, and 2.6/year for Marsh sites when focusing just on the upper 11 cm of soil [32]; these estimates would have a higher concentration of fine roots that likely grow and die quicker than coarser roots between 11 and 30 cm deep (Fig 5). We found it very difficult to differentiate fine and coarse root size class among dead roots. It was clear that many dead coarse roots were cross-classifying as fine because they lost volume during fairly rapid decomposition on these field sites, so we abandoned the effort to separate. New roots lost 15–37% of their biomass over the first year on our forested sites versus 12–13% for marshes [25]. Thus, root turnover and longevity apply to classified fine and coarse roots in our study, but with the caveat that most of our coarse roots (and some that would classify as rhizomes) are < 5 mm in diameter and classified as fine by some accounts [34].

As TFFW transitions to marsh, including all roots make sense because we are comparing between habitats that potentially differ significantly in the types of roots used for nutrient acquisition. While most uptake of nutrients is likely to occur within smaller size classes ≤ 2 mm, based on modeling [48] and measurement [49] insight, we do not know exactly how this might differentiate between TFFW and marsh. Indeed, where significant correlations emerged within specific rivers between BNPP and porewater nutrients, they only occurred for marsh sites with a larger suite of classified coarse roots. NO_x_ did not relate to any estimate of BNPP when evaluated across all sites, but DRP and NH_3_ did (Fig 4). It was curious that only the shorter-term estimations of BNPP related to porewater nutrients in any way (either 6-month ingrowth or Max-Min calculations from serial coring), indicating that utilization of porewater nutrients is opportunistic or seasonal. Root longevity was 0.7–3.2 years among all of our techniques, calculation methods, and sites (Table 4), meaning that on average the volume of all classified fine and coarse roots from a particular technique assessment is produced and dies within that rather short period of time.

The higher salinities in Lower Forest sites versus Middle and Upper Forest sites were associated with higher soil total nitrogen [20], a potential consequence of forest degradation as leaves and fine woody debris fall to the soil and physiologically stressed trees lose capacity to re-cycle nitrogen [20, 26, 50]. Nitrogen mineralization also peaks on the Lower Forest sites for both rivers [30]. At this transition and into the nearby marshes, herbaceous plants can consume and likely proliferate from increased nitrogen; a process that should be clear from our BNPP data. In fact, along the Waccamaw/Sampit Rivers, the presence of NH_3_ significantly influenced BNPP for the marsh, but not for the adjacent Lower Forest site (Fig 4B). However, for the Savannah River, P mineralization, in lieu of N mineralization, was much higher in the Marsh [30], where the greater availability of DRP from this mineralization led to greater BNPP as estimated through 6-month ingrowth for the Savannah River marsh site (Fig 4E). Higher DRP also influenced Waccamaw/Sampit River BNPP positively (Fig 4A and 4C), indicating the potential importance of phosphorus biogeochemistry in mediating BNPP on transitional sites.

### The role of BNPP in total ecosystem productivity in retreating freshwater forests

When we regress aboveground herbaceous productivity from all sites [51] with BNPP [*sensu*, ref. 52], the relationship is significant with 6-month ingrowth BNPP estimates (r^2^ = 0.847; *P* = 0.007), and both herbaceous productivity and total aboveground productivity (incl. herbaceous and woody growth) are significant for 12-month ingrowth BNPP estimates (r^2^ = 0.721; *P* = 0.018). No significance was noted for serial coring estimations of BNPP. This may indicate that ingrowth techniques encompass herbaceous productivity from the upper soil layers over shorter-term deployments fairly well (6 months or less), where recovery of cut roots might be quicker among the suite of annual and perennial herbaceous plants versus woody plants. This may also mean that aboveground and belowground productivity was less coupled than we typically assume over these assessment periods.

From our root ingrowth assays, BNPP to a soil depth of 30-cm was slightly higher than what has been reported from other forested wetland ecosystems. However, sample depths (and root sizes) for fine-root BNPP are not standardized widely among the literature, and we sampled moderately deep in comparison with the forested wetland BNPP literature (S1 Table). Root BNPP to a depth of 30 cm from upper, middle, and lower forests from our root ingrowth assays averaged 141–359 g/m^2^/year, 175–783 g/m^2^/year, and 419–1585 g/m^2^/year, respectively, with marsh BNPP averaging 895–1650 g/m^2^/year. BNPP of fine roots (≤ 3 mm) to a depth of 10.77 cm was 153.9, 181.0, and 93.7 g/m^2^/year, respectively, for well-drained, intermediately drained, and poorly drained floodplain forests along the Coosawhatchie River, South Carolina, USA [53]. For a mixed hardwood site within the Great Dismal Swamp, Virginia, root BNPP (including ≤ 2 mm, 2–5 mm, and > 5 mm roots) to a depth of 40 cm was up to 989 g/m^2^/year, but this rate of productivity was not maintained among other habitat types in the same study, which included Atlantic white cedar (*Chamaecyparis thyoides*) wetlands (up to 366 g/m^2^/year), baldcypress wetlands (up to 308 g/m^2^/year), and red maple-blackgum (*Nyssa sylvatica*) wetlands (up to 59 g/m^2^/year) [54]. BNPP of fine roots (≤ 5 mm) to a depth of 20 cm was 190–455 g/m^2^/year for clear-cut and intact swamp tupelo, sweetbay (*Magnolia virginiana*), and red maple wetlands along a small stream in Alabama, USA [55]. Additional BNPP estimates from floodplain and mangrove wetlands from the Southeastern United States expand the range of literature values to 59–989 g/m^2^/year, which canvass depths from the soil surface down to 11–45 cm deep (S1 Table). Of note, our BNPP estimations from serial coring were far greater than these literature values from forested wetlands.

One issue potentially not specific to all forested wetlands relates to clustered spatial variability in BNPP. For example, Jones et al. [55] investigated BNPP differences among microtopographic location (i.e., small mounds at the base of trees, or hummocks; depressional areas between trees, or hollows), and found that fine root BNPP was up to nearly 5 times higher in certain months in hollows versus hummocks. Hummock and hollow microtopography is a prominent feature among TFFW of the southeastern US [56], including both of our fresh Upper Forest sites. All of our BNPP data were taken from areas classified as “hollows”, potentially elevating our fine root BNPP estimates in light of what Jones et al. [55] found. In contrast, TBCA approaches would include both topographic positions spatially, depending upon how components of the TBCA calculations are estimated [24]. However, this microtopographic differentiation in BNPP is also not a consistent result; fine root BNPP (< 2 mm) to a depth of 20 cm averaged 455 g/m^2^/year for a mixed hardwood site along the Alligator River in North Carolina, USA, with 77% of that BNPP associated with hummocks [57]. Production of absorptive roots (i.e., first and second order roots) contributed more to hummock BNPP than transport roots (i.e., third-order roots), with transport roots contributing proportionately higher to BNPP in hollows [57]. We did not differentiate root order, but the extreme values of specific cores from individual study sites (Fig 3) indicate that more effort to differentiate the spatial nature of root growth in fresh and low salinity tidal forested wetlands is warranted.

## Conclusions

As hypothesized, estimation of BNPP in low salinity tidal wetlands is influenced by sampling technique and/or calculation method applied. Root ingrowth appears to be an adequate estimator of BNPP in forested wetlands with fewer herbaceous plants, and serial coring accounts for more components of BNPP as rhizomatous marsh species invade forested wetlands. Different BNPP approaches impose artifacts through damage during sampling, or accentuate growth preferences into root-free media versus root-occupied soil. As these artifacts can be stratified between forest and marsh (grasslands), BNPP techniques applied need consideration, as ensemble approaches as we use here are not often feasible. That specific BNPP techniques related to porewater nutrient availability on some sites, while other BNPP techniques did not, is noteworthy. Having multiple assessments and rates of BNPP from the same sites using different approaches may be useful for future process-driven modeling efforts (e.g., model calibration and validation) and for understanding the role played by the rhizosphere of retreating freshwater forests in mediating nutrient acquisition and soil volume expansion.

## Supporting information

S1 Fig Four-month hydrograph for Waccamaw/Sampit river study sites. Hydrographs are for (A) Upper Forest, (B) Middle Forest, and (C) Lower Forest (blue) and Marsh (pink) belowground net primary productivity (BNPP) study sites for the period 1 December 2013 to 31 March 2014 comparing the relative hydrology of each study site relative to the hummock bottoms (y-axis = 0 cm), where BNPP cores were extracted/inserted.
(TIF)

S2 Fig Four-month hydrograph for Savannah river study sites. Hydrographs are for (A) Upper Forest, (B) Middle Forest, and (C) Lower Forest (blue) and Marsh (pink) belowground net primary productivity (BNPP) study sites for the period 1 December 2013 to 31 March 2014 comparing the relative hydrology of each study site relative to the hummock bottoms (y-axis = 0 cm), where BNPP cores were extracted/inserted.
(TIF)

S3 Fig Belowground net primary productivity (BNPP, median) for TFFW and marshes by technique across all sites. BNPP was estimated using root ingrowth techniques over 6 months, root ingrowth techniques over 12 months, Smalley estimation of serial coring data, Maximum-Minimum (Max-Min) estimation of serial coring data, and total belowground carbon allocation (TBCA) for data collected from TFFW transitioning to marsh along the Waccamaw/Sampit Rivers, South Carolina, and the Savannah River, Georgia.
(TIF)

S1 Table Estimates of belowground net primary productivity (BNPP) from forested wetlands of the Southeastern United States. Data are reported by location, forested wetland (Site) type, dominant tree species, BNPP estimation method, soil depth of assessment, and reference (from main text).
(XLSX)
