# Supplementary figures and images for: Belowground productivity varies by assessment technique, vegetation type, and nutrient availability in tidal freshwater forested wetlands transitioning to marsh
Source: PLoS One. 2021 Jul 16;16(7):e0253554. doi: 10.1371/journal.pone.0253554 (PMC8284669; doi:10.1371/journal.pone.0253554)

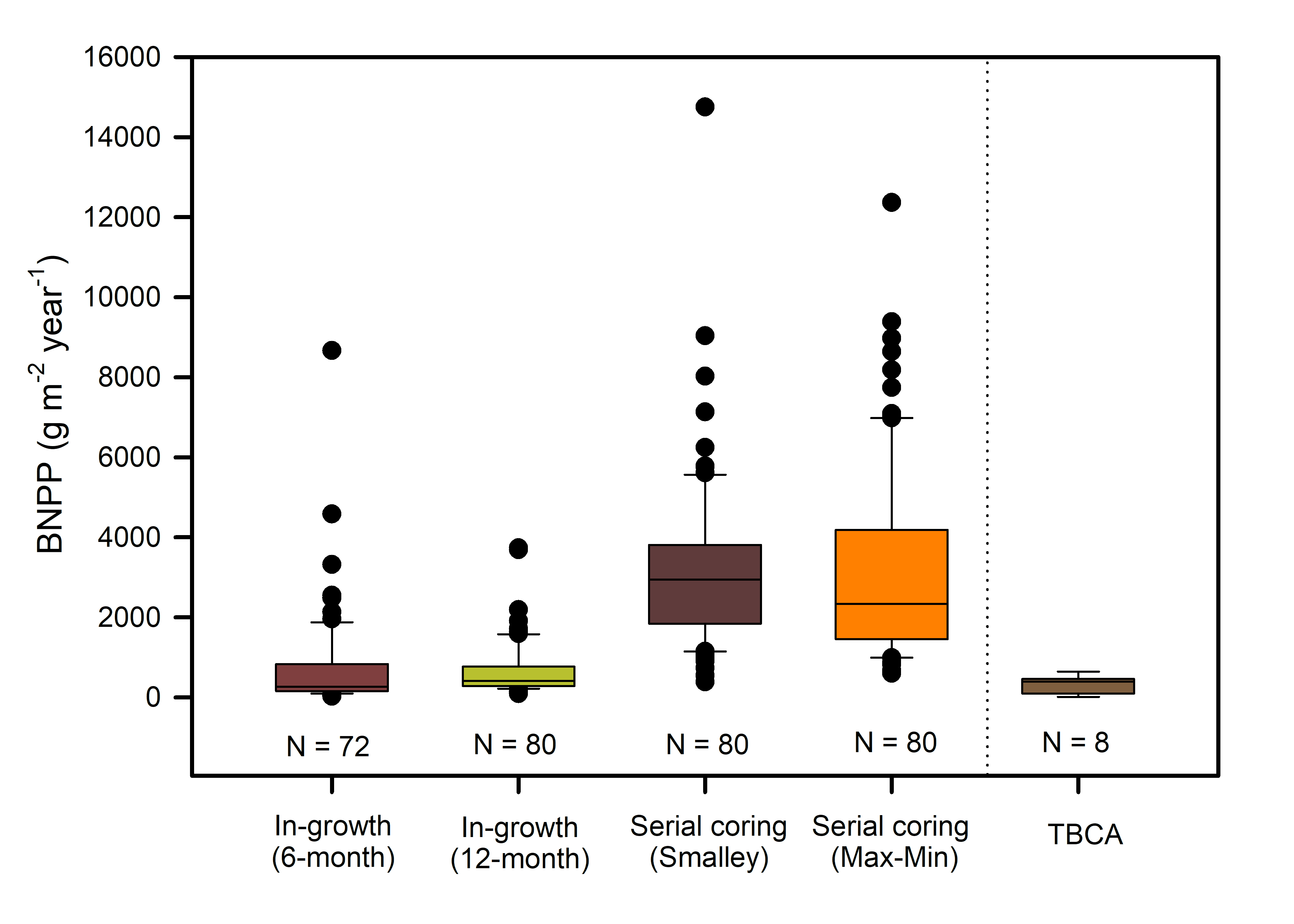

Supplement: S3 Fig — BNPP was estimated using root ingrowth techniques over 6 months, root ingrowth techniques over 12 months, Smalley estimation of serial coring data, Maximum-Minimum (Max-Min) estimation of serial coring data, and total belowground carbon allocation (TBCA) for data collected from TFFW transitioning to marsh along the Waccamaw/Sampit Rivers, South Carolina, and the Savannah River, Georgia. (TIF) [file pone.0253554.s003.tif]
